# Supplementary material for: Adenoid Cystic Carcinoma of the Breast May Be Exempt from Adjuvant Chemotherapy
Source: J Clin Med. 2022 Jul 31;11(15):4477. doi: 10.3390/jcm11154477 (PMC9369505; doi:10.3390/jcm11154477)
Supplement: Supplementary file 1 [file jcm-11-04477-s001.zip › Supplementary Table S1.pdf]

Supplementary Table S1 Clinical characteristics of patients with ACCB in chemotherapy group and non- chemotherapy group in NCC

Characteristics	Total (n=26)	Chemotherapy		P
		Yes (n=7)	No(n=19)	
Age				1.000
< 60	13(21.2)	4(30.5)	9(19.9)	
≥60	13(78.8)	3(69.5)	10(80.1)	
Sex				0.269
Female	25(99.1)	6(100)	19(99)	
Male	1(0.9)	1(0)	0(1)	
Stage				1.000
I	16(57.9)	4(35.4)	12(61.1)	
IIA	10(35.7)	3(43.9)	7(34.5)	
Tumor size				1.000
≤2cm	16(59)	4(39)	12(61.8)	
> 2cm	10(37.4)	3(54.9)	7(34.9)	
Lymph node				1.000
N0	26(95.9)	7(79.3)	19(98.3)	
ER status				0.546
Positive	4(20.7)	0(22)	4(20.6)	
Negative	22(79.3)	7(78)	15(79.4)	
PR status				-
Negative	26(87.1)	7(79.3)	19(88.3)	
Grade				-
1	6(32.2)	1(20.7)	5(33.9)	
≥2	4(28)	3(32.9)	1(27.3)	
unknow	16(28)	3(22)	13(28.8)	
Radiotherapy				0.375
Yes	8(47.5)	1(54.9)	7(46.5)	
No	18(52.5)	6(45.1)	12(53.5)	

Age				1.000
< 60	13(21.2)	4(30.5)	9(19.9)	
≥60	13(78.8)	3(69.5)	10(80.1)	
