# Supplementary figures and images for: Adenoid Cystic Carcinoma of the Breast May Be Exempt from Adjuvant Chemotherapy
Source: J Clin Med. 2022 Jul 31;11(15):4477. doi: 10.3390/jcm11154477 (PMC9369505; doi:10.3390/jcm11154477)

## Distribution of Propensity Scores

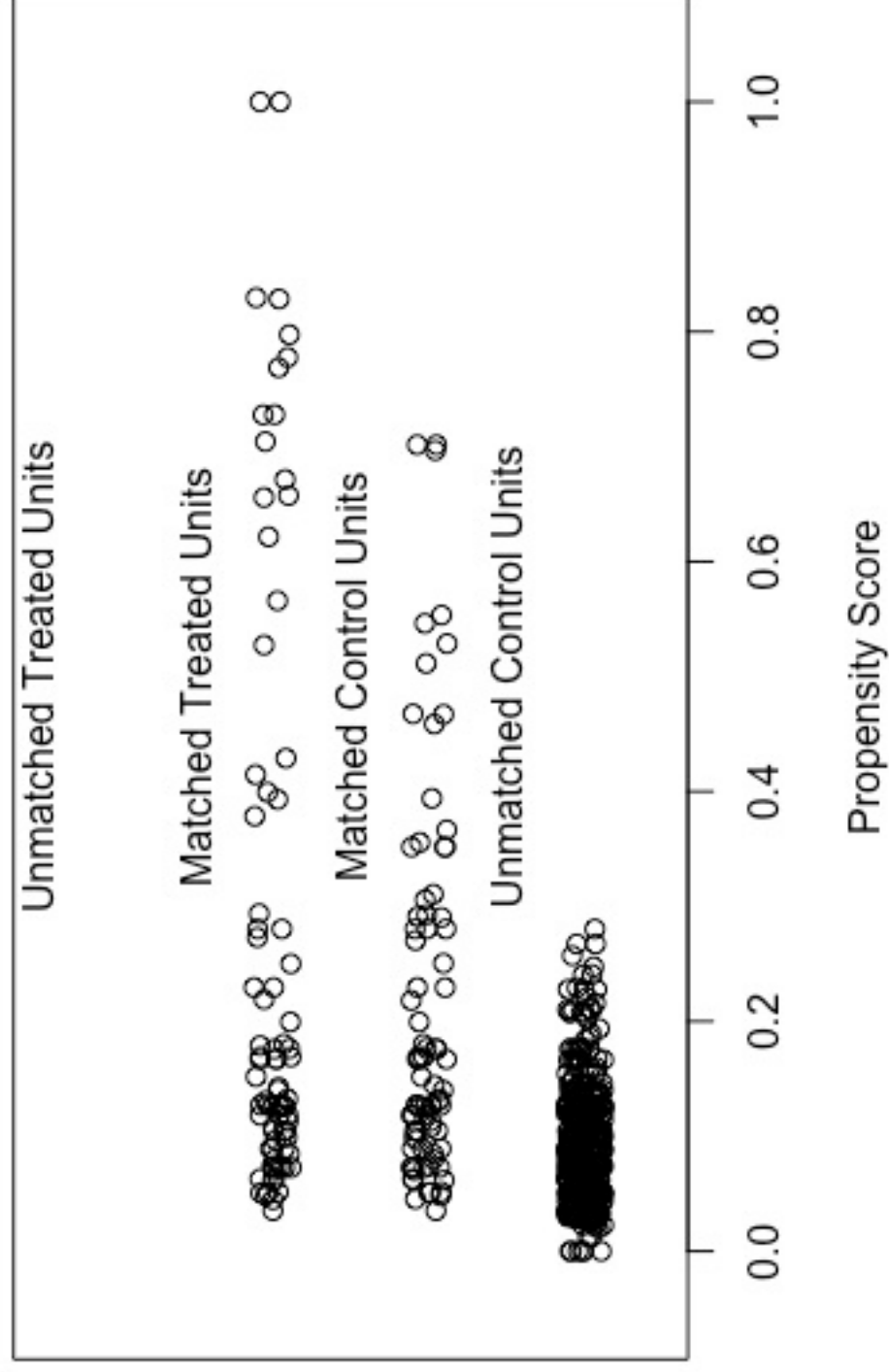

Supplement: Supplementary file 1 [file jcm-11-04477-s001.zip › Supplementary Figure S1.pdf]

**Raw Treated**

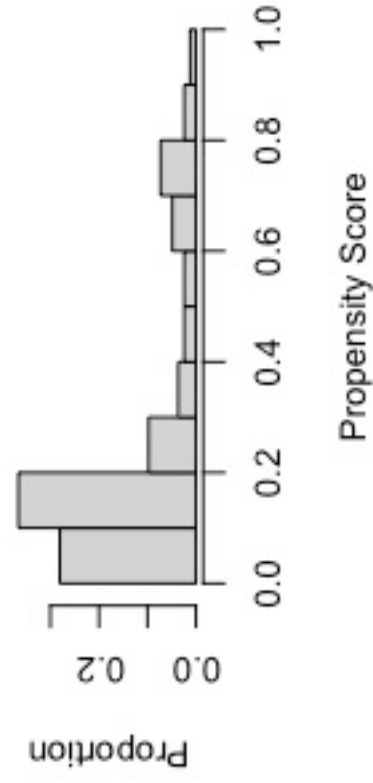

**Matched Treated**

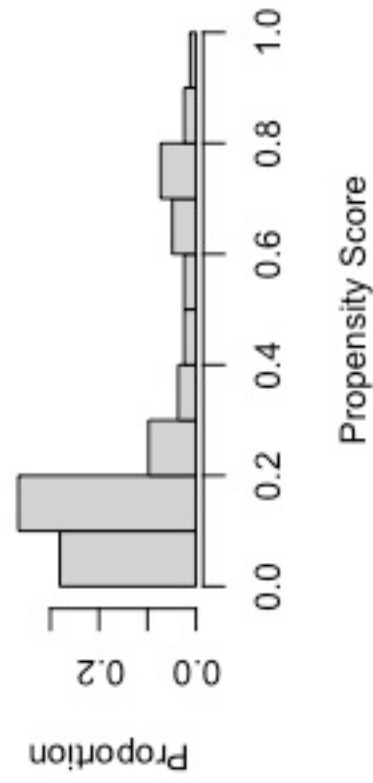

**Raw Control**

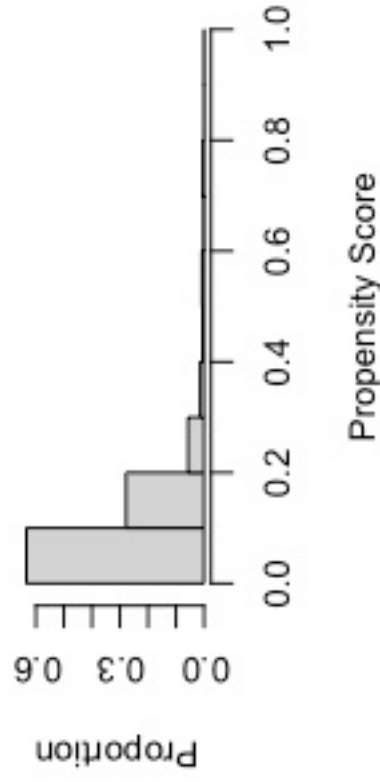

**Matched Control**

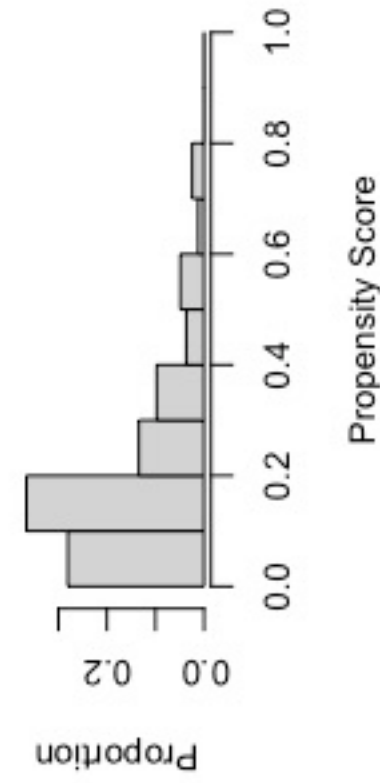

Supplement: Supplementary file 1 [file jcm-11-04477-s001.zip › Supplementary Figure S2.pdf]

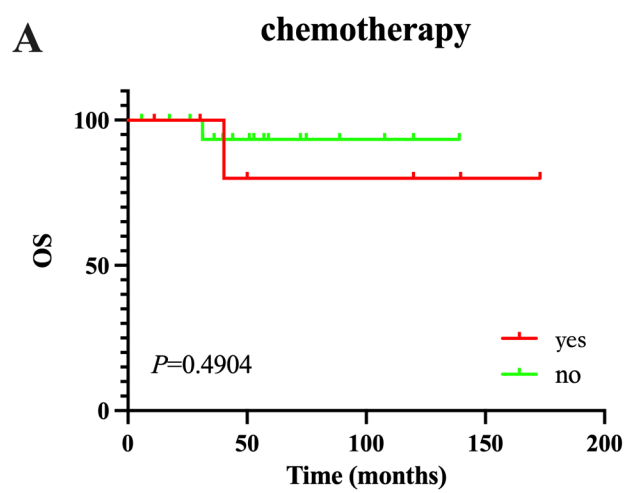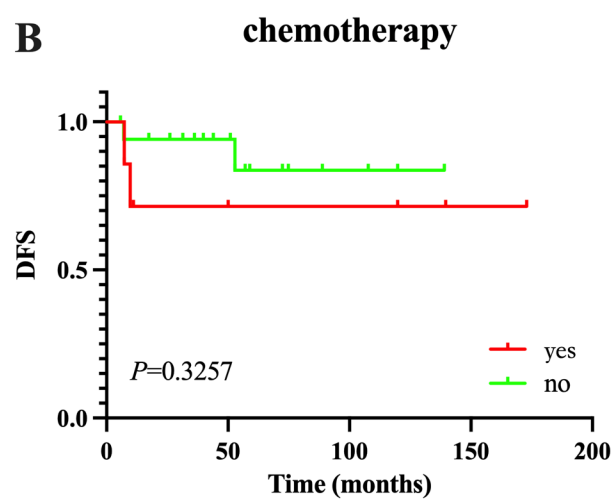

Supplement: Supplementary file 1 [file jcm-11-04477-s001.zip › Supplementary Figure S3.pdf]
